# Application of Nested PCR-DGGE (Denaturing Gradient Gel Electrophoresis) for the Analysis of Ciliate Communities in Soils

**DOI:** 10.1264/jsme2.ME11287

**Published:** 2011-12-01

**Authors:** Satoshi Shimano, Mitsuo Sambe, Yasuhiro Kasahara

**Affiliations:** 1 Miyagi University of Education, Aramaki-aza-Aoba, Aoba-ku, Sendai 980–0845, Japan; 2 Institute of Low Temperature Science, Hokkaido University, Kita 19, Nishi 8, Kita-ku, Sapporo 060–0819, Japan

**Keywords:** 18S rRNA gene, ciliate community, nested PCR-DGGE, soil

## Abstract

Ciliates play important roles as prey and predators in ecosystems. Changes in the ciliate community can affect the composition and population of microfauna and microflora in ecosystems. To investigate the structure of ciliate communities, we developed a nested PCR-DGGE method, which combines a universal eukaryotic-specific primer set in the first PCR step with a ciliate-specific primer set in the second PCR step, to amplify 18S rRNA genes from ciliates. The 300 bp DGGE fragments generated more bands on the gel than the 600 bp DGGE fragments. Prior to bead beating, DNA extraction of ciliates from soil samples was optimized with a combination of freeze-thaw cycles and ultrasonication. We applied this nested PCR-DGGE method to agricultural soils amended with 0, 120, 300, and 600 t ha^−1^ year^−1^ of livestock slurry. The results from the DGGE profiles and principal component analysis (PCA) revealed that the supplement of slurry to soils influenced the ciliate communities. From phylogenetic analysis, 108 DGGE bands were assigned to six classes, which included *Spirotrichea* and *Colpodea*, of the subphylum *Intramacronucleata*, and one class of the subphylum *Postciliodesmatophora*. These results indicated that a wide variety of taxonomic groups were detected by DGGE profiling. Thus, the nested PCR-DGGE method described here could clearly differentiate between ciliate communities within soil samples and allowed for the phylogenetic identification of these ciliates at the class level.

Ciliates (Phylum *Ciliophora*) play important roles in terrestrial ecosystems. They are a key link in the food web because they are major predators of bacteria (10, 20), as well as prey for larger organisms (12, 13). Ciliates are also known to stimulate bacterial mineralization of nutrients (2, 15) and to regulate microbial biomass (31) and the composition of bacterial communities. Consequently, ciliates have direct and indirect effects on energy and nutrient flows.

The population of ciliates in soil is estimated to be between 100 and 18,000 cells per gram of soil (6, 33). The most abundant soil ciliates have short generation times and the ability to multiply rapidly in response to increases in bacterial production (31); therefore, to identify ciliates, the use of conventional methods, which include morphological examinations of fixed and stained cells with light microscopy and scanning electron microscopy of cultured cells, are unsuitable for analyses of many samples taken during brief fluctuations in the compositions of ciliate communities.

Accurate ecological research of microorganisms requires the application of molecular techniques: PCR-DGGE (denaturing gradient gel electrophoresis) is a popular technique for analyzing changes in microbial diversity (23) due to its ease of handling, high-throughput format, time efficiency and easily understandable visual output that does not require laborious processes, such as clonal sequencing or culturing. The DGGE method has been used to analyze eukaryotic communities of samples from the ocean (5), activated sludge (19), rice field soil (21), and lake water (34). The application of taxon-specific primers with DGGE has been used for the identification of protists (4, 11, 26), fungi (1, 3, 14), and nematodes (35).

There are a few reports on the diversity of ciliate communities using DGGE; soil polluted with polycyclic aromatic hydrocarbons (PHA) (16); Oligotrich and Choreot-rich ciliates in a large temperate estuary (30); and in the rumen of domestic sheep, deer, and cattle (18). For the aforementioned studies, the PCR-DGGE protocols varied in a number of parameters: primer sequences, primer sets, targeting regions, amplicon sizes and thermal cycle profiles. In this study, we developed a nested PCR-DGGE method for the analysis of soil ciliate communities, and optimized the extraction of DNA from ciliates in soil by combining freeze-thaw cycles and sonication. Finally, to test the performance of our PCR-DGGE method, we examined the community structures of ciliates from soils with variations in applied livestock slurry.

## Materials and Methods

### Soil

Soil samples were collected from the upper 5 cm of an agricultural field that was supplemented with 0, 120, 300, and 600 t ha^−1^ year^−1^ of livestock slurry at the National Agricultural Research Center for the Kyushu Okinawa Region, Miyazaki prefecture, Japan in May 2005 and 2006. This agricultural field has been managed under similar conditions over time, including the application of supplemental slurry (24). Crops have been rotated between maize (April to August) and Italian ryegrass (September to March) since 1985.

### Ciliate cells

We have previously isolated five ciliate strains of the order *Sporadotrichina* (St03: *Oxytricha granulifera*, St05: *Gonostomum strenuum*, St09: *Pattersoniella vitiphila*, St13: *Oxytricha granulifera*, and St20: *Oxytricha* sp.), and one ciliate strain of the order *Stichotrichida* (St04: *Orthoamphisiella breviseries*) from soil from Daikoku-jima Island, Hokkaido, Japan (28), and one strain of *Levicoleps biwae* (order *Prorodontida* [*Levicoleps biwae*]) from mud on the shore of Lake Biwa (10). The cells of these ciliate strains were stored in sterile Milli-Q water at −20°C until PCR amplification.

### DNA extraction from soils

Soil was filtered through a 5 mm mesh and total DNA was extracted from soil samples (0.5 g). The effect of freeze-thaw and sonication procedures was tested in all possible combinations prior to using ISOIL for Bead Beating (Nippon Gene, Tokyo, Japan). In the freeze-thaw procedure, samples were first frozen rapidly by immersion in liquid nitrogen for 2 min and were then subsequently thawed in a 60°C water bath for 5 min. This entire freeze-thaw process was repeated three times. The sonication procedure was conducted at 100 W for 5 min in an ultrasonicator (VS-F100; AS One, Tokyo, Japan). DNA extraction was performed in triplicate and total DNA yield was calculated from the absorbance at 260 nm using a UV spectrophotometer (Ultrospec 2100pro; GE Healthcare, Little Chalfont, UK)

### Nested PCR

All of the primers used in the study are presented in Table 1. The 18S rRNA gene fragments were amplified with primers EU60F and EU929R for the first PCR step, and either CS322F (25) and EU581RGC or CS322F and EU929RGC sets for the second nested PCR. The reverse primers used for nested PCR included a GC clamp. The template DNA used for PCR amplification consisted of a single cell of the ciliate or the soil DNA extracts diluted 10 times in sterile water. Each 50 μL PCR mixture contained 5 μL of 10× Ex Taq buffer, 4 μL of 0.25 mM dNTP mixture, 1 μL of 50 pmol μL^−1^ of each primer, 1.25 U of *Ex Taq* DNA polymerase (Takara, Otsu, Japan) and template DNA, which was a single ciliate cell or 1 μL diluted DNA extract. The PCR amplification conditions were: 95°C denaturation for 5 min, followed by 25 touchdown cycles of 94°C for 1 min, 65°C (0.5°C decreased per cycle) for 30 s, 72°C for 1 min, followed by 10 cycles of 94°C for 1 min, 55°C for 30 s, and 72°C for 30 s, with a final extension at 72°C for 5 min. Nested PCR was performed with one cycle at 95°C for 1 min, followed by the same thermal profile described above for the first PCR step. All PCR products were subsequently verified with 1.0% agarose gel electrophoresis and ethidium bromide staining.

### DGGE

PCR products were purified with a Wizard SV Gel and PCR Clean-Up System (Promega, Madison, WI, USA), and quantified with a UV spectrophotometer. DGGE was performed as described by Muyzer *et al.*(22) using the D-code system (Bio-Rad Laboratories, Hercules, CA, USA). DNA samples (150 ng) were loaded onto an 8% polyacrylamide gel, which was made with a denaturing gradient in the range of 30%–60%. The denaturant (100%) contained 7 M urea and 40% formamide. Electrophoresis was run in 1 × TAE buffer at 60°C for 16 h at 75 V. After electrophoresis, gels were stained with SYBR Gold (Invitrogen, Carlsbad, CA, USA) and subsequently photographed under UV transillumination using a charge-coupled device camera (Image Server; Atto, Tokyo, Japan). A DGGE Marker II (Nippon Gene) was used as a molecular weight marker.

### Sequencing and phylogenetic analysis

Nucleotide sequencing was performed using an ABI BigDye Terminator v3.1 Cycle Sequencing Kit and an ABI 3730xl sequencer (Applied Biosystems, Carlsbad, CA, USA) according to the manufacturer’s instructions. The 18S rRNA gene fragments from DGGE bands were sequenced with CS322F and EU581R primers and approximately 270 bp were sequenced on a single strand. The partial 18S rRNA gene sequences from the DGGE bands were compared with database sequences using nucleotide-nucleotide BLAST (BLASTN) as a means to obtain the nearest phylogenetic neighbors (www.ncbi.nlm.nih.gov/BLAST/). Trees were constructed from libraries obtained by the neighbor joining method within the program MEGA 5 (29) and bootstrapped with 1,000 repetitions.

### Statistical analysis

The variation in total DNA yield and number of DGGE bands for the four combinations of freeze-thaw and/or sonication procedures were assessed using Tukey’s multiple comparison test. Principal component analysis (PCA) of the PCR-DGGE profiles was performed to elucidate the ciliate community structures based on the relative band intensity and positions using BioNumerics (Applied Maths NV, Sint-Martens-Latem, Belgium). This software also identified bands in the same position in different lanes of the gel and measured the intensity of the bands identified from digitized DGGE images.

### Accession numbers of nucleotide sequences

The nucleotide sequences obtained in this study were deposited in the DDBJ database with the following accession numbers: AB646993 to AB647106.

## Results and Discussion

### Examination of the nested PCR-DGGE method

Separation of DGGE bands is extremely important to distinguish between single base-pair changes of DGGE fragments. DGGE profiles were used to amplify the 18S rRNA gene fragment by simple PCR using the CS322F/EU581RGC and CS322F/EU929RGC primer sets as templates of soil environmental DNA. The results of DGGE did not show any band (only smear band) in each lane (data not shown); therefore we developed a nested PCR-DGGE method.

**Fig. 1:**
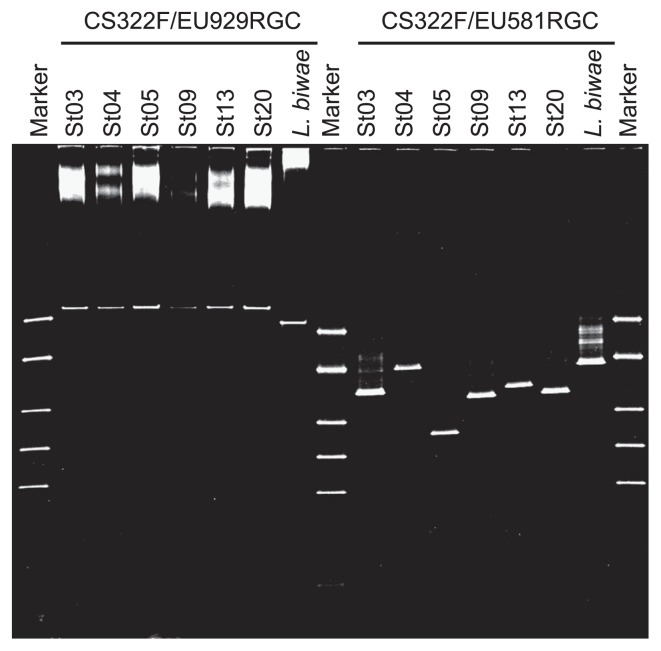
DGGE profiles of partial 18S rRNA genes amplified with nested-PCR using CS322F/EU581RGC and CS322F/EU929RGC primer sets from different single cells of order *Sporadotrichina* (St03: *Oxytricha granulifera*, St05: *Gonostomum strenuum*, St09: *Pattersoniella vitiphila*, St13: *O. granulifera*, and St20: *Oxytricha* sp.), order *Stichotrichia* (St04: *Orthoamphisiella breviseries*), and *L. biwae* (order *Prorodontida* [*L. biwae*]).

To examine DGGE profiles, the 18S rRNA gene fragments of single ciliate cells were first amplified using the EU60F/EU929R primer set, which provided template DNA for the second amplification with nested PCR using two different reverse primer sets, CS322F/EU581RGC and CS322F/EU929RGC. DGGE profiles from five strains of *Sporadotrichina*, one strain of *Stichotrichia* and *L. biwae* that were generated using two primer sets, CS322F/EU581RGC and CS322F/EU929RGC, are shown in Fig. 1. In the DGGE profile derived from the CS322F/EU929RGC set, all bands from six strains of *Sporadotrichina* and *Stichotrichia* were in the same location and were indistinguishable, whereas the bands using the CS322F/EU581RGC set migrated to four distinct locations: St03 and St13, St04, St05, and St09 and St20. In the amplified region from the CS322F/EU581RGC set (256 bp excluding the GC clamp), the DNA sequence of St03 and St13, and of St09 and St20 were identical. The St03 DNA sequence had 1 bp, 5 bp, and 10 bp mismatches relative to St09 and St20, St05, and St04, respectively. The DGGE band locations could be divided into four groups by DNA sequence. The region from the CS322F/EU581RGC set was shown to provide a higher resolution DGGE profile that clearly reflected the sequence variation. These results indicated that the nested PCR-DGGE method using the primer set CS322F/EU581RGC is more effective than the CS322F/EU929RGC set for separating groups of ciliates from a DNA sample.

**Fig. 2:**
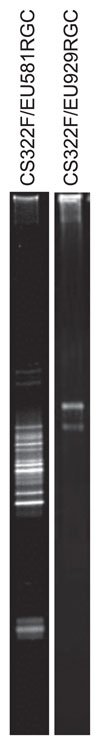
DGGE profiles of 18S rRNA genes amplified with nested-PCR using the CS322F/EU581RGC and CS322F/EU929RGC primer sets in the second PCR from soil DNA.

To evaluate the resolution for the DGGE profiles with two different reverse primer sets for the soil samples, the ciliate community in the soil supplemented with 600 t ha^−1^ of livestock slurry from 2005 was analyzed (Fig. 2). The DGGE profile with the EU581RGC reverse primer exhibited 17 bands, whereas the DGGE profile with the EU929RGC primer had two bands. Both profiles differed markedly between the amplified regions. The amplicon sizes for the CS322F/EU581RGC and CS322F/EU929RGC sets were approximately 300 bp and 620 bp, respectively. The short DNA fragment yielded a higher resolution DGGE profile. Generally, in PCR-DGGE analysis of microbial communities, a forward primer with a GC clamp is used; thus, we examined DGGE profiles for using a GC clamp with the forward primer CS322F. Consequently, many bands did not separate and were unresolved (data not shown). The use of reverse primers with a GC clamp improved the DGGE banding pattern. These results suggest that the nested PCR-DGGE method using the CS322F/EU581RGC primer set is able to effectively analyze soil ciliate communities.

### Comparison of different treatments for soil DNA extraction

Ciliates pass through various cell forms during their life cycles, one of which is an encysted form. The cyst form provides resistance to environmental stress. Ciliates often enter soil cavities and pores and exploit bacterial food sources (8), inhabiting not only the surface, but also the interior of soil aggregates; therefore, physical treatments, such as freeze-thaw or sonication, are necessary for optimum performance due to their ability to destroy soil aggregates and lyse ciliate cells prior to DNA extraction.

A comparison of the freeze-thaw and sonication procedures using soil with 600 t ha^−1^ of slurry from 2005 indicated that neither treatment resulted in the highest DNA yield, and the combined treatment was the lowest of all (Table 2). The soil treatment significantly (P <0.01) affected DNA yield. The DGGE profile of the 300 bp fragment (based on the results in Fig. 2) from the ciliate community and each of the extracted DNAs is shown in Fig. S1. In the samples treated with both freeze-thaw cycles and sonication, we found more DGGE bands, although the differences from the other treatments were not significant (P <0.05, Table 2). These data may indicate that the combination of freeze-thaw treatment plus sonication enhanced the destruction of soil aggregates and ciliate cells. Consequently, the use of DNA extracts from soil treated with both freeze-thaw and sonication was more effective for analysis of the ciliate community.

### Application of DGGE method to agricultural soils

**Fig. 3:**
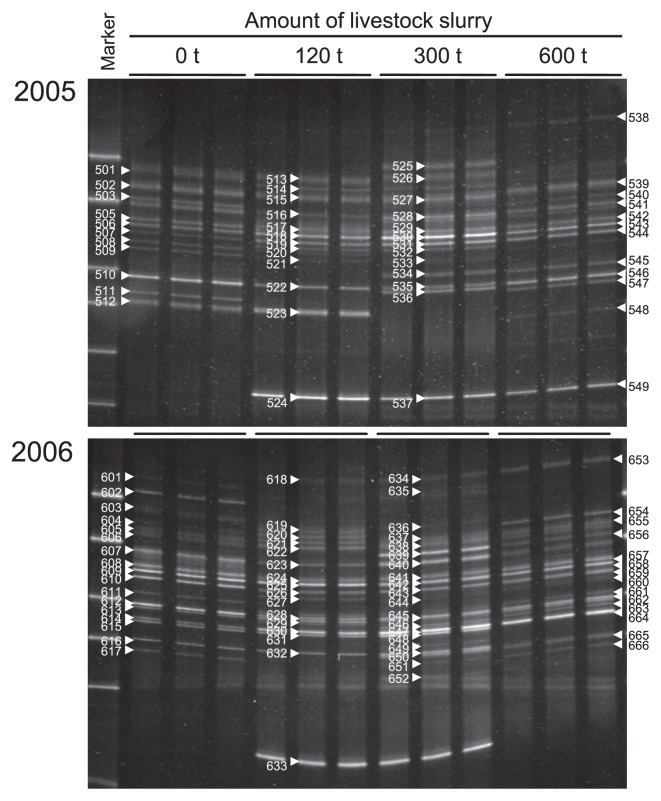
DGGE profiles of ciliate 18S rRNA genes from soils samples of each field supplemented with slurry in 2005 and 2006. Numbers indicate the sample name of DGGE bands for sequencing.

**Fig. 4:**
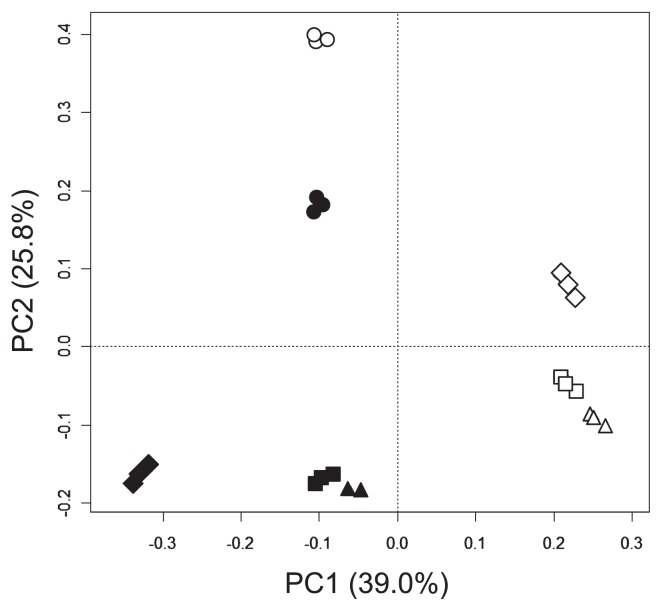
Principal component analysis (PCA) of the DGGE profiles of ciliate DNA from soil supplemented with livestock slurry 0 t (○ ●), 120 t (△ ▲), 300 t (□ ■), and 600 t ha^−1^ year^−1^ (⋄ ♦) in 2005 (open) and 2006 (closed). Percentages on axes denote the amount of variance explained by each principal component.

We applied the nested PCR-DGGE method to structural analysis of ciliate communities from agricultural soils supplemented with 0, 120, 300, and 600 t ha^−1^ of livestock slurry in 2005 and 2006. The DGGE profiles generated from each soil consisted of many bands (Fig. 3). There were 11–14 and 14–18 bands in the profiles from 2005 and 2006, respectively. The DGGE profiles were different in various slurry soils between the two years and no correlation was found between the number of bands and the amount of slurry. The number of bands did not necessarily increase with an increase in the supplement of slurry. A PCA plot based on these DGGE profiles confirmed the differences among the various slurry soils in the two years tested (Fig. 4). The soil samples from 2005 and 2006 had different ciliate communities. The 0 tha^−1^ soil samples were separated clearly from the other soils in both years. These data showed that the supplement of slurry to soils influenced the ciliate community. Nutrients, microorganisms and ciliates in the slurry may directly or indirectly affect indigenous microorganisms and ciliates in the soil. As a result, supplying slurry to soils affected the composition and proportion of the ciliate community.

A total 114 bands, 501–548 and 601–666, in the DGGE profiles from 2005 and 2006, respectively, were selected for phylogenetic analysis (Fig. 3). Of these 114 bands, 108 bands belonged to *Ciliophora*, and the remaining 6 bands (SS_502, SS_512, SS_513, SS_515, SS_527, and SS_634) belonged to other eukaryotic groups (Table S1). About 94.7% of the selected bands were 18S rRNA genes from ciliates that were amplified using the nested PCR-DGGE method with the CS322F/EU581RGC primer set. In the phylogenetic tree, 56, 33, 3, 2, 1, and 1 band were affiliated with class *Spirotrichea*, *Colpodea*, *Litostomatea*, *Oligohymenophorea*, *Phyllopharyngea*, and *Heterotrichea*, respectively (Fig. S2). The SS_635 band was associated with subphylum *Intramacronucleata* class *Phyllopharyngea*, and the SS_649 band was associated with subphylum *Postciliodesmatophora* class *Heterotrichea*. These specific SS_635 and SS_649 bands were only observed in 300 t ha^−1^ slurry soil from 2006. In the same soil sample, *Labyrinthula*, which belongs to *Stramenopiles* and not *Ciliophora*, was detected (SS_634 band), which is interesting because this organism is ordinarily found in sea environments. The reason for the detection of this ciliate from soil is not clear. Twelve bands detected from this analysis (SS_524, SS_537, SS_549, SS_631, SS_633, SS_637, SS_643, SS_650, SS_651, SS_652, SS_663 and SS_665) formed a novel clade (Group I in Fig. S2) and might be affiliated with a new class.

To optimize DNA extraction from soils for ciliate community analysis, a treatment using freeze-thaw and sonication was used, which destroys not only ciliate cells, but also other eukaryotic cells, such as other protists, fungi, nematode, and arthropods. From sequence analysis, it was determined that almost all of the 18S rRNA gene fragments obtained with our nested PCR-DGGE method originated from ciliate cells, indicating that the second primer sets were highly specific to *Ciliophora*. Although the lengths of the obtained fragments were short (approximately 260 bp), the DGGE profiles were higher resolution and thus were able to reveal differences in ciliate communities between the soil samples. Due to the low variation between DNA sequences used in the alignments, the classification from the base of the phylogenetic tree was at the class taxonomic level (Fig. S2).

In conclusion, we recommend our reliable method for the analysis of soil ciliate communities. This method involves DNA extraction with a combination of freeze-thaw cycles plus ultrasonication treatments prior to bead beating of soil samples. Amplification of soil samples is accomplished with two PCR steps, one standard PCR with a universal eukaryotic primer, followed by one nested PCR with a ciliate-specific primer set (CS322F/EU581RGC); and subsequent separation and visualization with DGGE.

## Supplemental material



## Figures and Tables

**Table 1:** Primers used in this study

Primer	Target	Sequence (5′–3′)^a^	Position^b^	Reference
EU60F	Eukaryote	GAAACTGCGAATGGCTCATT	79–98	36
CS322F	*Ciliophora*	GATGGTAGTGTATTGGAC	313–330	25
EU581R	Eukaryote	ATTACCGCGGCTGCTGGC	557–574	36
EU581RGC	Eukaryote	GC clamp-ATTACCGCGGCTGCTGGC	557–574	36
EU929R	Eukaryote	TTGGCAAATGCTTTCGC	913–929	36
EU929RGC	Eukaryote	GC clamp-TTGGCAAATGCTTTCGC	913–929	36

a GC clamp: CGCCCGCCGCGCCCCGCGCCGGTCCCGCCGCCCCCGCCCG

b Corresponding position in the SSU rRNA gene in *Tetrahymena corlissi* (U17356)

**Table 2:** Effect of different soil treatments on DNA extraction and DGGE analysis

Treatments Freeze-thaw/Sonication^a^	DNA yield^b^ (μg g^−1^soil)	No. of DGGE bands
+/+	16.2±1.4 a	20.0±0.8
+/−	19.3±1.1 b	17.7±2.1
−/+	17.6±1.1 a	14.3±1.7
−/−	20.8±0.9 c	14.7±2.6

All values are the means ± SD (*n*=3).

a +, with treatments; −, without treatment.

b Different letters indicate a significant difference at P <0.01 (Tukey’s multiple comparison test).
