# Supplementary material for: Application of Nested PCR-DGGE (Denaturing Gradient Gel Electrophoresis) for the Analysis of Ciliate Communities in Soils
Source: Microbes Environ. 2011 Dec 1;27(2):136–41. doi: 10.1264/jsme2.ME11287 (PMC4036011; doi:10.1264/jsme2.ME11287)
Supplement: Supplementary file 1 [file 27_136_s1.pdf]

Supplemental material

Fig. S1. DGGE profiles from soil samples using freeze-thaw and/or sonication.

Fig. S2. Phylogenetic analysis showing the relationship between DGGE band of 18S rRNA gene amplicons and their nearest neighbors. The tree is based on a final alignment of 257 bases. The distance scale indicates 0.02 substitutions/site. Bootstrapping analysis of 1,000 replicates was performed using ClustalW (bootstrap values >50% are given). Sample name of DGGE band is indicated by a number (DGGE band name is SS_*).

Table S1. List of the identification of DGGE bands.

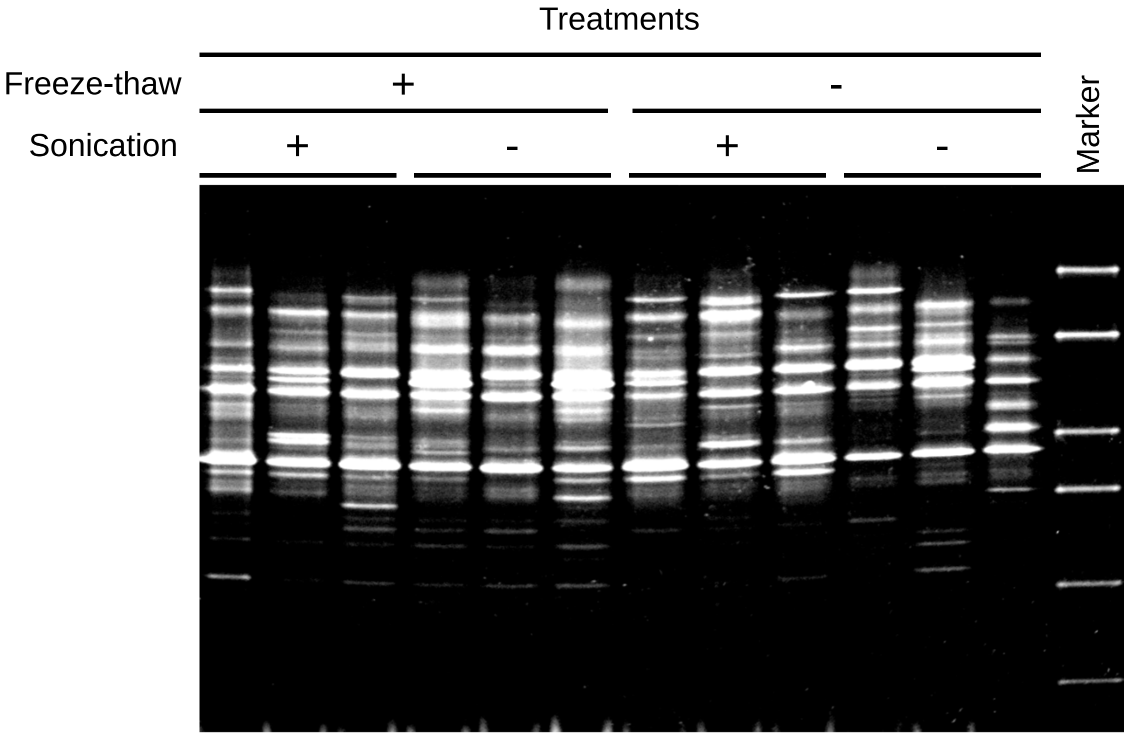

Fig. S1.

Shimano S. et al.

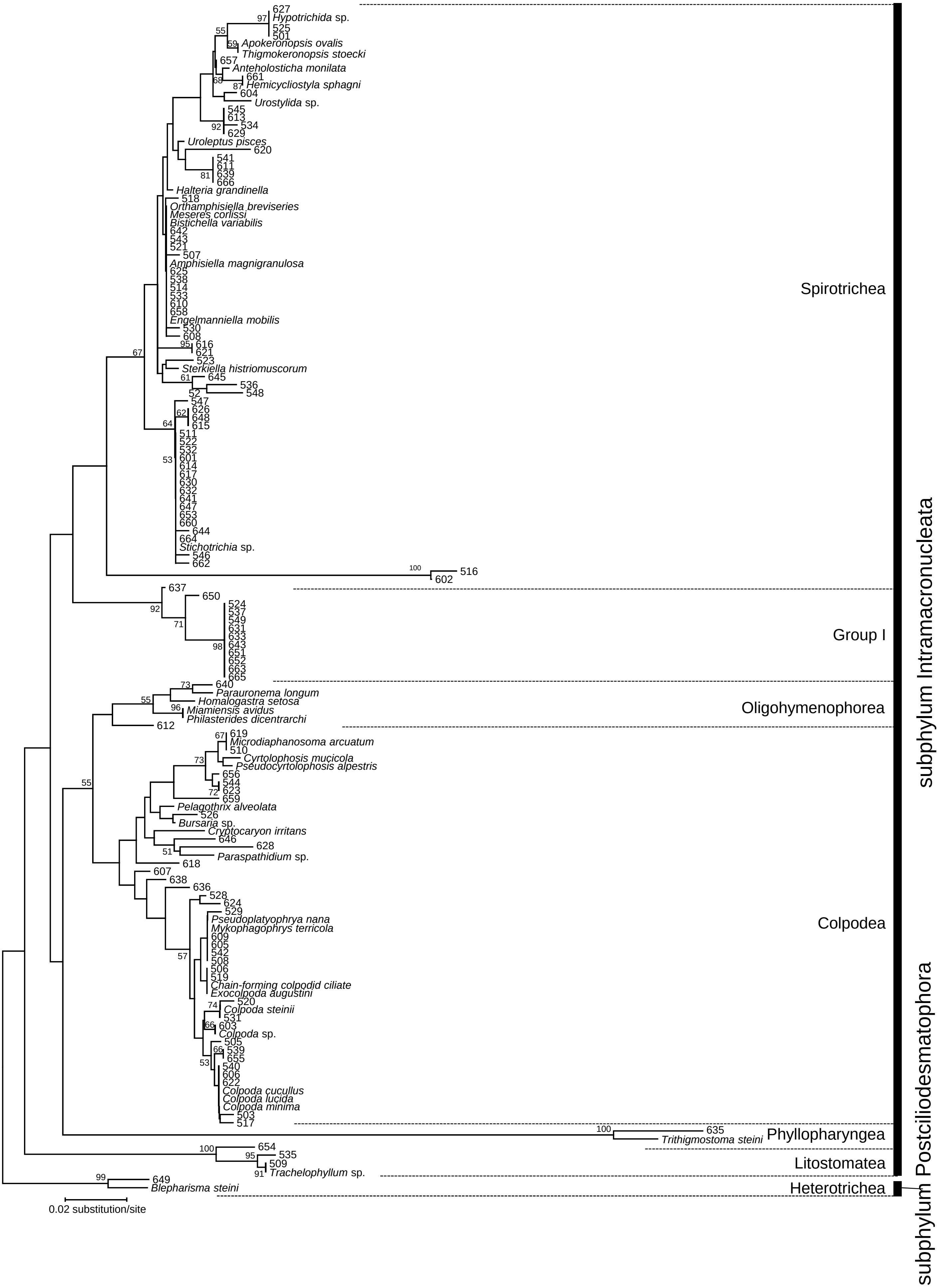

Fig. S2.

Shimano S. et al.

Table S1

Soil sample	Band no.	Accession no.	Closest match (accession no.)	Similarity	Phylogenetic group
2005 0 t	SS_501	AB646993	*Hypotrichida* sp. (DQ022066)	99%	Alveolata; Ciliophora; Intramacronucleata; Spirotrichea
	SS_502	AB646994	*Paracercomonas crassicauda* (FJ790725)	98%	Rhizaria; Cercozoa; Cercomonadida; Cercomonadida
	SS_503	AB646995	*Colpoda minima* (EU039897)	99%	Alveolata; Ciliophora; Intramacronucleata; Colpodea
	SS_505	AB646996	*Colpoda steinii* (DQ388599)	99%	Alveolata; Ciliophora; Intramacronucleata; Colpodea
	SS_506	AB646997	*Bistichella variabilis* (HQ699895)	99%	Alveolata; Ciliophora; Intramacronucleata; Spirotrichea
	SS_507	AB646998	*Bistichella variabilis* (HQ699895)	99%	Alveolata; Ciliophora; Intramacronucleata; Spirotrichea
	SS_508	AB646999	*Exocolpoda augustini* (JF747214)	99%	Alveolata; Ciliophora; Intramacronucleata; Colpodea
	SS_509	AB647000	*Trachelophyllum* sp. (JF263452)	99%	Alveolata; Ciliophora; Intramacronucleata; Litostomatea
	SS_510	AB647001	*Microdiaphanosoma arcuatum* (GU997633)	98%	Alveolata; Ciliophora; Intramacronucleata; Colpodea
	SS_511	AB647002	*Stichotrichia* sp. (AB449362)	99%	Alveolata; Ciliophora; Intramacronucleata; Spirotrichea
	SS_512	AB647003	*Labyrinthula* sp. (AB290459)	92%	Stramenopiles; Labyrinthulida; Labyrinthulidae
120 t	SS_513	AB647004	*Paracercomonas producta* (FJ790721)	98%	Rhizaria; Cercozoa; Cercomonadida; Cercomonadida
	SS_514	AB647005	*Bistichella variabilis* (HQ699895)	100%	Alveolata; Ciliophora; Intramacronucleata; Spirotrichea
	SS_515	AB647006	Soil flagellate AND18 (AY965864)	98%	Rhizaria; Cercozoa
	SS_516	AB647007	*Mykophagophrys terricola* (EU039902)	99%	Alveolata; Ciliophora; Intramacronucleata; Colpodea
	SS_517	AB647008	*Colpoda lucida* (EU039895)	99%	Alveolata; Ciliophora; Intramacronucleata; Colpodea
	SS_518	AB647009	*Amphisiella magnigranulosa* (AM412774)	99%	Alveolata; Ciliophora; Intramacronucleata; Spirotrichea
	SS_519	AB647010	Chain-forming colpodid ciliate (AY398684)	99%	Alveolata; Ciliophora; Intramacronucleata; Colpodea
	SS_520	AB647011	*Colpoda steinii* (DQ388599)	99%	Alveolata; Ciliophora; Intramacronucleata; Colpodea
	SS_521	AB647012	*Engelmanniella mobilis* (AF508757)	100%	Alveolata; Ciliophora; Intramacronucleata; Spirotrichea
	SS_522	AB647013	*Stichotrichia* sp. (AB449362)	99%	Alveolata; Ciliophora; Intramacronucleata; Spirotrichea
	SS_523	AB647014	*Sterkiella histriomuscorum* (HQ615720)	99%	Alveolata; Ciliophora; Intramacronucleata; Spirotrichea
	SS_524	AB647015	*Philasterides dicentrarchi* (GU572375)	94%	Alveolata; Ciliophora; Intramacronucleata; Oligohymenophorea
300 t	SS_525	AB647016	*Hypotrichida* sp. (DQ022066)	100%	Alveolata; Ciliophora; Intramacronucleata; Spirotrichea
	SS_526	AB647017	*Bursaria* sp. (EU039889)	99%	Alveolata; Ciliophora; Intramacronucleata; Colpodea
	SS_527	AB647018	*Paracercomonas crassicauda* (FJ790725)	96%	Rhizaria; Cercozoa; Cercomonadida; Cercomonadida
	SS_528	AB647019	*Mykophagophrys terricola* (EU039902)	99%	Alveolata; Ciliophora; Intramacronucleata; Colpodea
	SS_529	AB647020	*Pseudoplatyophrya nana* (AF060452)	99%	Alveolata; Ciliophora; Intramacronucleata; Colpodea
	SS_530	AB647021	*Bistichella variabilis* (HQ699895)	99%	Alveolata; Ciliophora; Intramacronucleata; Spirotrichea
	SS_531	AB647022	*Colpoda steinii* (DQ388599)	100%	Alveolata; Ciliophora; Intramacronucleata; Colpodea
	SS_532	AB647023	*Stichotrichia* sp. (AB449362)	99%	Alveolata; Ciliophora; Intramacronucleata; Spirotrichea
	SS_533	AB647024	*Stichotrichia* sp. (AB449364)	100%	Alveolata; Ciliophora; Intramacronucleata; Spirotrichea
	SS_534	AB647025	*Thigmokeronopsis stoecki* (EU220226)	98%	Alveolata; Ciliophora; Intramacronucleata; Spirotrichea
	SS_535	AB647026	*Enchelyodon* sp. (JF263446)	99%	Alveolata; Ciliophora; Intramacronucleata; Litostomatea
	SS_536	AB647027	*Sterkiella histriomuscorum* (HQ615720)	98%	Alveolata; Ciliophora; Intramacronucleata; Spirotrichea
	SS_537	AB647028	*Philasterides dicentrarchi* (GU572375)	94%	Alveolata; Ciliophora; Intramacronucleata; Oligohymenophorea
600 t	SS_538	AB647029	*Bistichella variabilis* (HQ699895)	100%	Alveolata; Ciliophora; Intramacronucleata; Spirotrichea
	SS_539	AB647030	*Colpoda lucida* (EU039895)	99%	Alveolata; Ciliophora; Intramacronucleata; Colpodea
	SS_540	AB647031	*Colpoda cucullus* (EU039893)	100%	Alveolata; Ciliophora; Intramacronucleata; Colpodea
	SS_541	AB647032	*Meseres corlissi* (EU399529)	98%	Alveolata; Ciliophora; Intramacronucleata; Spirotrichea
	SS_542	AB647033	*Mykophagophrys terricola* (EU039902)	100%	Alveolata; Ciliophora; Intramacronucleata; Colpodea
	SS_543	AB647034	*Bistichella variabilis* (HQ699895)	100%	Alveolata; Ciliophora; Intramacronucleata; Spirotrichea
	SS_544	AB647035	*Cyrtolophosis mucicola* (EU039898)	98%	Alveolata; Ciliophora; Intramacronucleata; Colpodea

Table S1

	SS_545	AB647036	*Thigmokeronopsis stoecki* (EU220226)	98%	Alveolata; Ciliophora; Intramacronucleata; Spirotrichea
	SS_546	AB647037	*Stichotrichia* sp. (AB449362)	99%	Alveolata; Ciliophora; Intramacronucleata; Spirotrichea
	SS_547	AB647038	*Stichotrichia* sp. (AB449362)	99%	Alveolata; Ciliophora; Intramacronucleata; Spirotrichea
	SS_548	AB647039	*Stichotrichia* sp. (AB449362)	97%	Alveolata; Ciliophora; Intramacronucleata; Spirotrichea
	SS_549	AB647040	*Philasterides dicentrarchi* (GU572375)	94%	Alveolata; Ciliophora; Intramacronucleata; Oligohymenophorea
2006 0 t	SS_601	AB647041	*Stichotrichia* sp. (AB449362)	99%	Alveolata; Ciliophora; Intramacronucleata; Spirotrichea
	SS_602	AB647042	*Pseudocyrtolophosis alpestris* (EU264564)	99%	Alveolata; Ciliophora; Intramacronucleata; Colpodea
	SS_603	AB647043	*Colpoda* sp. (GQ475427)	100%	Alveolata; Ciliophora; Intramacronucleata; Colpodea
	SS_604	AB647044	*Urostylida* sp. (GU967698)	98%	Alveolata; Ciliophora; Intramacronucleata; Spirotrichea
	SS_605	AB647045	*Mykophagophrys terricola* (EU039902)	100%	Alveolata; Ciliophora; Intramacronucleata; Colpodea
	SS_606	AB647046	*Colpoda lucida* (EU039895)	100%	Alveolata; Ciliophora; Intramacronucleata; Colpodea
	SS_607	AB647047	*Mykophagophrys terricola* (EU039902)	97%	Alveolata; Ciliophora; Intramacronucleata; Colpodea
	SS_608	AB647048	*Bistichella variabilis* (HQ699895)	99%	Alveolata; Ciliophora; Intramacronucleata; Spirotrichea
	SS_609	AB647049	*Mykophagophrys terricola* (EU039902)	100%	Alveolata; Ciliophora; Intramacronucleata; Colpodea
	SS_610	AB647050	*Bistichella variabilis* (HQ699895)	99%	Alveolata; Ciliophora; Intramacronucleata; Spirotrichea
	SS_611	AB647051	*Meseres corlissi* (EU399529)	98%	Alveolata; Ciliophora; Intramacronucleata; Spirotrichea
	SS_612	AB647052	*Homalogastra setosa* (EF158848)	98%	Alveolata; Ciliophora; Intramacronucleata; Oligohymenophorea
	SS_613	AB647053	*Thigmokeronopsis stoecki* (EU220226)	98%	Alveolata; Ciliophora; Intramacronucleata; Spirotrichea
	SS_614	AB647054	*Stichotrichia* sp. (AB449362)	99%	Alveolata; Ciliophora; Intramacronucleata; Spirotrichea
	SS_615	AB647055	*Stichotrichia* sp. (AB449362)	99%	Alveolata; Ciliophora; Intramacronucleata; Spirotrichea
	SS_616	AB647056	*Meseres corlissi* (EU399529)	98%	Alveolata; Ciliophora; Intramacronucleata; Spirotrichea
	SS_617	AB647057	*Stichotrichia* sp. (AB449362)	99%	Alveolata; Ciliophora; Intramacronucleata; Spirotrichea
120 t	SS_618	AB647058	*Bursaria* sp. (JF747212)	97%	Alveolata; Ciliophora; Intramacronucleata; Colpodea
	SS_619	AB647059	*Microdiaphanosoma arcuatum* (GU997633)	98%	Alveolata; Ciliophora; Intramacronucleata; Colpodea
	SS_620	AB647060	*Uroleptus pisces* (AF164131)	98%	Alveolata; Ciliophora; Intramacronucleata; Spirotrichea
	SS_621	AB647061	*Meseres corlissi* (EU399529)	98%	Alveolata; Ciliophora; Intramacronucleata; Spirotrichea
	SS_622	AB647062	*Colpoda lucida* (EU039895)	100%	Alveolata; Ciliophora; Intramacronucleata; Colpodea
	SS_623	AB647063	*Cyrtolophosis mucicola* (EU039898)	98%	Alveolata; Ciliophora; Intramacronucleata; Colpodea
	SS_624	AB647064	*Pseudoplatyophrya nana* (AF060452)	99%	Alveolata; Ciliophora; Intramacronucleata; Colpodea
	SS_625	AB647065	*Bistichella variabilis* (HQ699895)	100%	Alveolata; Ciliophora; Intramacronucleata; Spirotrichea
	SS_626	AB647066	*Stichotrichia* sp. (AB449362)	99%	Alveolata; Ciliophora; Intramacronucleata; Spirotrichea
	SS_627	AB647067	*Hypotrichida* sp. (DQ022066)	99%	Alveolata; Ciliophora; Intramacronucleata; Spirotrichea
	SS_628	AB647068	*Cryptocaryon irritans* (AF351579)	95%	Alveolata; Ciliophora; unclassified Ciliophora
	SS_629	AB647069	*Apokeronopsis ovalis* (EU930048)	98%	Alveolata; Ciliophora; Intramacronucleata; Spirotrichea
	SS_630	AB647070	*Stichotrichia* sp. (AB449362)	99%	Alveolata; Ciliophora; Intramacronucleata; Spirotrichea
	SS_631	AB647071	*Philasterides dicentrarchi* (GU572375)	94%	Alveolata; Ciliophora; Intramacronucleata; Oligohymenophorea
	SS_632	AB647072	*Stichotrichia* sp. (AB449362)	98%	Alveolata; Ciliophora; Intramacronucleata; Spirotrichea
	SS_633	AB647073	*Miamiensis avidus* (EU831212)	94%	Alveolata; Ciliophora; Intramacronucleata; Oligohymenophorea
300 t	SS_634	AB647074	*Paracercomonas producta* (FJ790721)	98%	Rhizaria; Cercozoa; Cercomonadida; Cercomonadidae
	SS_635	AB647075	*Trithigmostoma steini* (X71134)	95%	Alveolata; Ciliophora; Intramacronucleata; Phyllopharyngea
	SS_636	AB647076	*Colpoda minima* (EU039897)	98%	Alveolata; Ciliophora; Intramacronucleata; Colpodea
	SS_637	AB647077	*Pelagothrix alveolata* (AB486009)	93%	Alveolata; Ciliophora; Intramacronucleata; Prostomatea
	SS_638	AB647078	*Colpoda cucullus* (EU039893)	98%	Alveolata; Ciliophora; Intramacronucleata; Colpodea
	SS_639	AB647079	*Halteria grandinella* (AF508759)	98%	Alveolata; Ciliophora; Intramacronucleata; Spirotrichea

Table S1

	SS_640	AB647080	*Paraurонema longum* (HM236338)	99%	Alveolata; Ciliophora; Intramacronucleata; Oligohymenophorea
	SS_641	AB647081	*Stichotrichia* sp. (AB449362)	99%	Alveolata; Ciliophora; Intramacronucleata; Spirotrichea
	SS_642	AB647082	*Orthamphisiella breviseries* (AY498654)	100%	Alveolata; Ciliophora; Intramacronucleata; Spirotrichea
	SS_643	AB647083	*Philasterides dicentrarchi* (GU572375)	94%	Alveolata; Ciliophora; Intramacronucleata; Oligohymenophorea
	SS_644	AB647084	*Stichotrichia* sp. (AB449362)	99%	Alveolata; Ciliophora; Intramacronucleata; Spirotrichea
	SS_645	AB647085	*Sterkiella histriomuscorum* (HQ615720)	99%	Alveolata; Ciliophora; Intramacronucleata; Spirotrichea
	SS_646	AB647086	*Paraspathidium* sp. (FJ875140)	97%	Alveolata; Ciliophora; Intramacronucleata; Litostomatea
	SS_647	AB647087	*Stichotrichia* sp. (AB449362)	99%	Alveolata; Ciliophora; Intramacronucleata; Spirotrichea
	SS_648	AB647088	*Stichotrichia* sp. (AB449362)	99%	Alveolata; Ciliophora; Intramacronucleata; Spirotrichea
	SS_649	AB647089	*Blepharisma steini* (AM713187)	98%	Alveolata; Ciliophora; Postciliodesmatophora; Heterotrichea
	SS_650	AB647090	*Philasterides dicentrarchi* (GU572375)	94%	Alveolata; Ciliophora; Intramacronucleata; Oligohymenophorea
	SS_651	AB647091	*Miamiensis avidus* (EU831212)	94%	Alveolata; Ciliophora; Intramacronucleata; Oligohymenophorea
	SS_652	AB647092	*Philasterides dicentrarchi* (GU572375)	94%	Alveolata; Ciliophora; Intramacronucleata; Oligohymenophorea
600 t	SS_653	AB647093	*Stichotrichia* sp. (AB449362)	99%	Alveolata; Ciliophora; Intramacronucleata; Spirotrichea
	SS_654	AB647094	*Fuscheria* sp. (JF263448)	99%	Alveolata; Ciliophora; Intramacronucleata; Litostomatea
	SS_655	AB647095	*Colpoda lucida* (EU039895)	99%	Alveolata; Ciliophora; Intramacronucleata; Colpodea
	SS_656	AB647096	*Pseudocyrtolophosis alpestris* (EU264564)	98%	Alveolata; Ciliophora; Intramacronucleata; Colpodea
	SS_657	AB647097	*Anteholosticha monilata* (GU942567)	99%	Alveolata; Ciliophora; Intramacronucleata; Spirotrichea
	SS_658	AB647098	*Bistichella variabilis* (HQ699895)	100%	Alveolata; Ciliophora; Intramacronucleata; Spirotrichea
	SS_659	AB647099	*Microdiaphanosoma arcuatum* (GU997633)	96%	Alveolata; Ciliophora; Intramacronucleata; Colpodea
	SS_660	AB647100	*Stichotrichia* sp. (AB449362)	99%	Alveolata; Ciliophora; Intramacronucleata; Spirotrichea
	SS_661	AB647101	*Hemicycliostyla sphagni* (FJ361758)	100%	Alveolata; Ciliophora; Intramacronucleata; Spirotrichea
	SS_662	AB647102	*Stichotrichia* sp. (AB449362)	99%	Alveolata; Ciliophora; Intramacronucleata; Spirotrichea
	SS_663	AB647103	*Philasterides dicentrarchi* (GU572375)	94%	Alveolata; Ciliophora; Intramacronucleata; Oligohymenophorea
	SS_664	AB647104	*Stichotrichia* sp. (AB449362)	99%	Alveolata; Ciliophora; Intramacronucleata; Spirotrichea
	SS_665	AB647105	*Philasterides dicentrarchi* (GU572375)	94%	Alveolata; Ciliophora; Intramacronucleata; Oligohymenophorea
	SS_666	AB647106	*Meseres corlissi* (EU399529)	98%	Alveolata; Ciliophora; Intramacronucleata; Spirotrichea
